# Optimizing Adaptive Notifications in Mobile Health Interventions Systems: Reinforcement Learning from a Data-driven Behavioral Simulator

**DOI:** 10.1007/s10916-021-01773-0

**Published:** 2021-10-18

**Authors:** Shihan Wang, Chao Zhang, Ben Kröse, Herke van Hoof

**Affiliations:** 1 Informatics Institute, University of Amsterdam, Amsterdam, Netherlands; 2 Information and Computing Sciences, Utrecht University, Utrecht, Netherlands; 3 Department of Psychology, Utrecht University, Utrecht, Netherlands; 4 Digital Life, Amsterdam University of Applied Sciences, Amsterdam, Netherlands; 5 Human-Technology Interaction, Eindhoven University of Technology, Eindhoven, Netherlands

**Keywords:** Mobile health intervention, Adaptive agent, Reinforcement learning, Human simulator, Just-in-time adaptive intervention

## Abstract

Mobile health (mHealth) intervention systems can employ adaptive strategies to interact with users. Instead of designing such complex strategies manually, reinforcement learning (RL) can be used to adaptively optimize intervention strategies concerning the user’s context. In this paper, we focus on the issue of overwhelming interactions when learning a good adaptive strategy for the user in RL-based mHealth intervention agents. We present a data-driven approach integrating psychological insights and knowledge of historical data. It allows RL agents to optimize the strategy of delivering context-aware notifications from empirical data when counterfactual information (user responses when receiving notifications) is missing. Our approach also considers a constraint on the frequency of notifications, which reduces the interaction burden for users. We evaluated our approach in several simulation scenarios using real large-scale running data. The results indicate that our RL agent can deliver notifications in a manner that realizes a higher behavioral impact than context-blind strategies.

**Supplementary Information:**

The online version contains supplementary material available at 10.1007/s10916-021-01773-0.

## Introduction

Adaptive interventions have emerged as a new perspective of prevention and treatment in healthcare [1]. The just-in-time adaptive intervention (JITAI) is an adaptive intervention design concept, aiming to provide the right type /amount of support at the right time based on an individual’s changing internal and external states [2, 3]. Though JITAIs can be administered through several means (e.g. in-person and computer), the ubiquity of mobile devices allows for continuous participant monitoring and delivery of personalized interventions. Mobile health systems (agents) with JITAIs have proven effective in preventing certain health threats (e.g. overeating [4], smoking [5] and prolonged sedentary behaviors [6]) and eliciting beneficial health outcomes (e.g. increased physical activity [7] and self-management support related to chronic diseases [8]). However, the design of such interventions is demanding and the interaction with the user can be complex. Reinforcement learning (RL) based agents have been used to optimize mobile healthcare interventions adaptively [9–11], which make use of historical data or data collected on the run. The problem of historical data is that it often misses counterfactual information (i.e. what would have been the outcome had interventions or circumstances been different). The problem of data collected during the intervention is that it requires many interactions in a short period, which add burden for the user and adversely impact engagement [12, 13].

Throughout the paper, we focus on optimizing the delivery of context-aware notifications in mobile health systems. These notifications are sent in an adaptive manner dependent on the temporal and environmental context of users, motivating them to perform a target activity. To solve the two mentioned problems, based on a framework that combines historical data and psychological theories about human decision-making, we developed a simulation environment to optimize the timing of these notifications. Moreover, to restrict interaction burden, we adapted an RL algorithm by incorporating a constraint on the number of notifications that can be sent within a period. Finally, we conducted a case study on promoting running activity to demonstrate our approach. A dataset covering over 10K real users’ running activity was used to build our simulator and evaluate our RL agent.

## Related work

For the optimization of JITAI intervention in mHealth systems, several different strategies were taken by researchers using RL [7, 8, 10, 11, 14]. However, most of those RL approaches require the agent to interact many times with the user before performing well. To shorten the online learning process, several researchers followed the concept of transfer learning to perform faster learning in mHealth settings. Tabatabaei et al. [15] and Tomkins et al. [16] make RL algorithms quickly learn from the limited experience at the beginning stage by considering similar users. Gonul et al. [17] transfer the common knowledge acquired in other environments to get faster convergence. Without constraints on the intervention frequency, those RL approaches might still bother users by too many interventions during fast learning. While they concentrate on using data collected during the online interventions, we follow another direction to solve this challenge, i.e., incorporating prior knowledge from historical data to optimize the policy in advance. Similar to our approach, Liao et al. [18] and Ameko et al. [19] integrate prior distributions using collected data in an RL optimization process. However, they apply relative small datasets in pre-learning because experimental data for specific intervention situations are often involving user interaction and therefore expensive to collect. Our framework allows learning prior knowledge from historical data collected without interacting with users, which makes the usage of large-scale data possible. To avoid many interactions in a short period, our approach for the first time performs a structural study to incorporate a constraint on interaction frequency in RL-based mHealth systems.

## Methodology

**Fig. 1:**
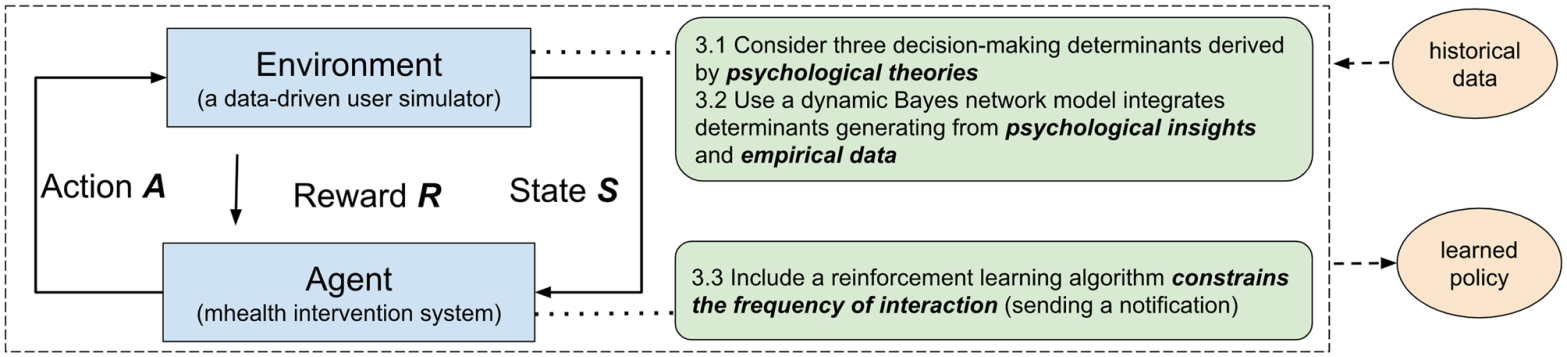
The overview of our methodology, including the agent–environment interaction in the MDP model and three key components developed in both environment and agent. The approach optimizes the delivery of context-aware notifications from empirical data

We model how users sequentially decide on whether to perform a target activity when receiving notifications (we use running as an example in this paper, in this case, the mobile agent sends notifications for promoting running activities). We formalize our problem (i.e. learning the optimal strategy for delivering notifications) as a finite horizon Markov Decision Process (MDP) [20]. Figure 1 presents an overview of our approach. Here, the agent represents a mobile system that interacts with a target user (i.e. the environment) to optimize the strategy. Our agent and environment interact in a sequence of discrete and finite time steps $\{1,2,..,t\}$, which can be naturally broken into episodes. At each time step, the agent observes a representation of the environment and selects an action accordingly (two possible actions in our case: send a notification or not send). The environment then passes a numerical reward back to the agent. Based on this feedback mechanism, the agent adapts its policy to maximize an expected long-term reward. Since too frequent interactions with the environment are not desirable in mHealth settings, we constrained the maximum number of notifications sent in each week (i.e. episode). In this paper, our optimization goal is to wisely deliver a restricted number of notifications to maximize the user’s weekly running frequency.

### Insight from psychological theories

Conceptually, it can be assumed that users’ decisions to engage in certain activities (e.g., running) after notifications take two steps, *option generation* and *option evaluation* [21–23]. At any decision moment, behavioral options have to be generated in memory before they can be compared to inform a final choice. Memory accessibility of different options during option generation is influenced by environmental cues, including system notifications. When a user receives a notification for running, the memory accessibility of running reaches its maximum. This accessibility then gradually decreases in the form of a memory decay until the next notification is received. The form of memory decay, or forgetting curve, is modeled as exponential functions in the psychology literature [22, 24].

After being generated, a target option (running) has to compete with other generated behavioral options (e.g. working on a paper) in terms of how much they satisfy a user’s personal goals, such as being healthy and productive. According to classic decision-making models [25, 26], the goal-satisfying values of options, weighted by the importance of the goals, are transformed into subjective utilities, and the option with the highest subjective utility will be chosen. Without enumerating all goal-related attributes, two types of attributes are important for running behavior. First, a user’s momentary context (e.g. time and weather) can have great impacts on decisions because the options’ goal-satisfying values depend on the contextual variables [27]. For example, a Sunday morning with good weather makes running more enjoyable and also less interfering with one’s work-related goals. Second, recently having a run ought to temporarily lower the utility of running. After a run, one’s body certainly needs time to recover to a level that is sufficient for running again. Furthermore, having a run satisfies running-related goals and attenuates the importance of the goals. As people pursue multiple goals, this psychological mechanism allows people to switch to other goals and engage in behaviors that satisfy those goals (e.g. finishing a manuscript to be productive) [28]. In summary, three key determinants of running decision - *memory accessibility* of running, *urge* of running, and personal *context* - were derived from the above theories and included in our computational model.

### Computational model

We formalized the above procedure as a dynamic Bayesian network (DBN). As a probabilistic graphical model, the DBN considers a set of variables and their conditional dependencies over adjacent time steps [29]. In this way, we generated a stochastic human simulator to make decisions based on both contextual and cognitive states sequentially.

#### Representation and topology of the DBN

Following the psychological theories above, we defined five variables and their dependencies in our DBN as follows:

- $A_{t}$ represents whether an user decides to take a target activity (running) at time *t*.
- $M_{t}$ is the user’s memory accessibility of running at time *t*.
- $U_{t}$ is the user’s urge to run at time *t*.
- $C_{t}$ is the personal context of the user at time *t*.
- $N_{t}$ represents whether the user receives a notification at time *t*.

**Fig. 2:**
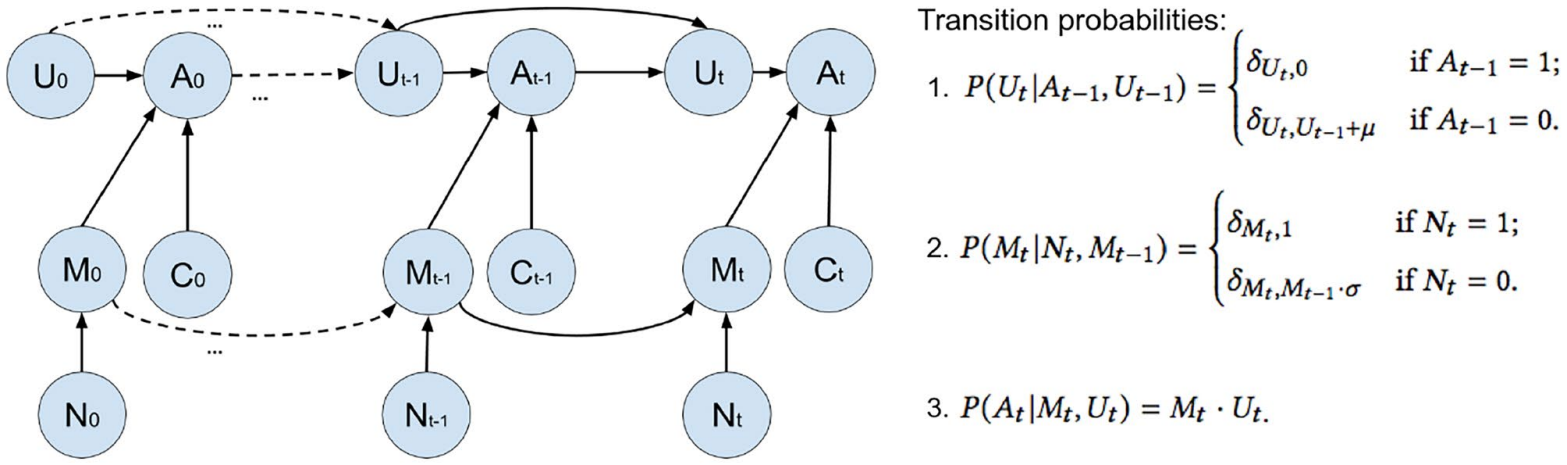
Topological structure and transition probabilities of our dynamic Bayesian network

The variable $M_{t}$ and $U_{t}$ are real values in (0, 1). The variable $N_{t}$ and $A_{t}$ are binary values $\in \{0,1\}$ , where ‘1’ represents ‘receive a notification’ and ‘decide to run’ respectively. The variable $C_{t}$ includes a set of contextual features, defined as a vector of values. Under the first-order Markov assumption, we proposed a topological structure of the DBN, as shown in Fig. 2.

#### Definitions and inference of the DBN

We specified transition probabilities in the DBN from either empirical data or psychological insights. Based on Insight from Psychological Theories, the state transitions of $U_{t}$ and $M_{t}$ were defined as Eqs. 1 and 2 in Fig. 2, where the notation $\delta$ represents the Kronecker delta function [30]. Given a certain $A_{t-1}$ and $N_{t}$, we deterministically have $U_{t}$ and $M_{t}$. The parameters $\mu$ and $\sigma$ define the changing rate of *urge* and *memory accessibility*. While memory accessibility decreases exponentially, the urge to run increases linearly over time. We also defined the transition from a joint observation of $M_{t}$ and $U_{t}$ to a target activity $A_{t}$ as Eq. 3 in Fig. 2. In particular, we proposed to calculate two probabilities $P(C_{t})$ and $P(C_{t} | A_{t})$ from empirical data (for details, see Data Description and Processing). Given these probabilities, we used the following equation to estimate how a user reacts to notifications.

1 $$\begin{aligned}P(A_{t}|M_{0\cdots t-1}, U_{0 \cdots t-1}, C_{0 \cdots t}, N_{0 \cdots t}) =& \ P(A_{t}|C_{t}, N_{t}, A_{t-1}, M_{t-1}, U_{t-1}) \\=& \sum _{M_{t}} \sum _{U_{t}} \frac{P(C_{t}| A_{t})}{P(C_{t})} \cdot P(A_{t}| U_{t}, M_{t}) \cdot P(M_{t}| N_{t}, M_{t-1}) \cdot P(U_{t}| A_{t-1}, U_{t-1})_{.} \end{aligned}$$

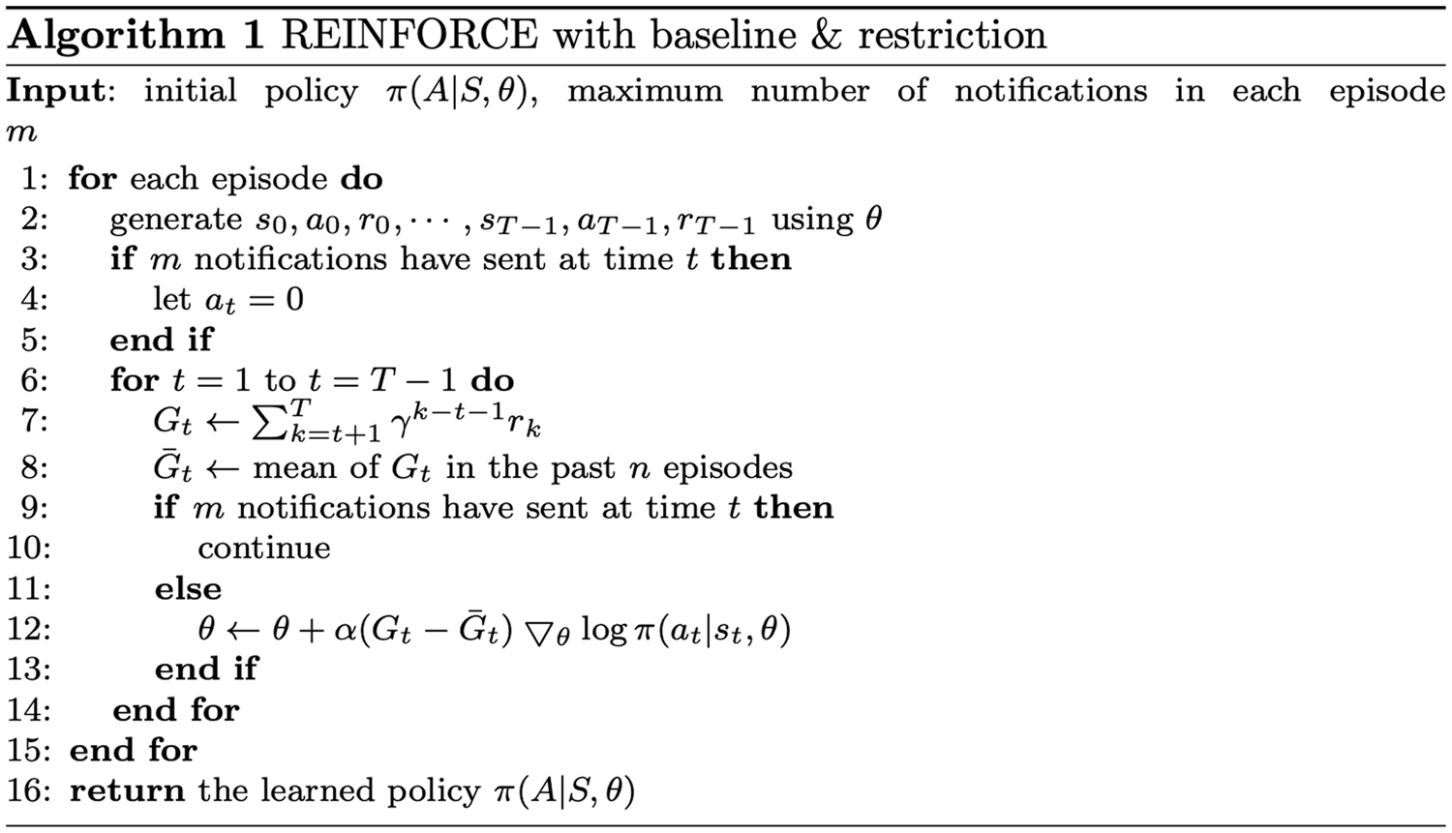


### Reinforcement learning algorithm

To learn the optimal policy (i.e. a stochastic mapping between a personal state of the user and an action to take) in our restricted setting, we adopted a policy gradient RL algorithm, REINFORCE [31]. The REINFORCE algorithm with baseline and restriction is outlined in Algorithm 1. Our algorithm updates based on episodes. In each episode, it performs a gradient step on a neural network to optimize the policy parameter $\theta$. We inserted a baseline function $\bar{G}_{t}$ inside the expectation to reduce the high variance, using the average of all returns $G_{t}$ in the past *n* episodes. Moreover, to integrate with the restricted setting, we adjusted the procedures of action selection and policy adaptation in the REINFORCE algorithm. Inspired by clipping the continuous action space in policy gradient [32], we constrained the probability of certain discrete actions. After reaching the maximum number of notifications in each episode, the probability of sending a notification is always 0. In this way, we make sure our RL algorithm learns to deliver a restricted number of notifications according to the given momentary state.

## Simulation experiments using real data

We demonstrated the performance of our approach in a case study, aiming at promoting running activities by sending context-aware notifications. Our approach was evaluated in a simulation environment using real running data.

### Experimental data and settings

#### Data description and processing

We used two datasets to derive the context related distributions in Eq. 1. First, a running dataset was used to derive the distribution $P(C_{t}|A_{t})$, measuring the relation between user context and running behavior. The data contains around 406K runs contributed by over 10K Dutch users while using a mobile fitness app from 2013-03 to 2017-03 [33]. For each run, a set of metadata is collected and timestamp and weather information at the beginning are marked. We considered six variables in the data, namely ‘hour of the day’, ‘weekday’, ‘temperature’, ‘weather type’, ‘wind type’ and ‘humidity type’. An example of context data is *{8:00, Monday, -2, cloudy, moderate wind, moderate humidity}*. Second, an open dataset provided by the Royal Netherlands Meteorological Institute (KNMI)^1^ was used to derive $P(C_{t})$, the prior distribution of contextual information (general Dutch weather), which contains around 439K records of hourly weather. To make the two datasets comparable, we used the weather data over the same period of the running data.

We derived distribution $P(C_{t}|A_{t})$ and $P(C_{t})$ from the running and the weather dataset in a same manner. Thus, we only demonstrate how we derived the context distribution from the running data. Since data are only available when a running activity is performed, we concentrate on computing the distribution $P(C_{t}|A_{t} = 1)$, which is a joint distribution of all contextual variables. Since we noticed that the feature ‘weekday’ is conditionally independent with other features, we learned the distribution $P(\text {weekday}_{t}| A_{t} = 1)$ by computing probabilities of all seven values in the categorized feature ‘weekday’. We also extracted the joint distribution of all the other features. For each combination of the discrete variables (weather, wind and humidity), we learned a separate multivariate Gaussian distribution for continuous variables (hour and temperature) using maximum likelihood estimation.

#### Setting of simulation with real contextual data

We implemented our simulation experiments using python^2^. The RL algorithm was developed based on pytorch^3^, and our RL agent and simulation environment were built following the framework of OpenAI gym^4^. In the simulation, the agent makes a decision on whether to send notification at every hour from 8:00 to 20:00. Only when the user performs a run before the next decision time step (within one hour), the agent gets a reward of 1.0 (otherwise zero reward). In our environment, each episode is one week and maximum of 14 notifications are allowed in each week. We also provided realistic context information in the simulation environment by using empirical data in the used KNMI dataset. Based on the results of a simulator verification^5^, we set memory retention rate ($\sigma$ in Eq. 2) at 0.8 and urge recovery rate ($\mu$ in Eq. 1) at 0.05. The discount factor $\gamma$ and learning rate $\alpha$ are set to 1 and 0.001 respectively. We ran each simulation 20 times. In each run, the environment starts at 0:00 of a ***random*** date with its corresponding real weather data.

### Experimental results

We evaluated our data-driven RL approach in two experiments. To set a comparable environment, we randomly initialize a single simulation environment for all agents of each experiment at every simulation run.

#### Evaluation of context-aware policy

**Fig. 3:**
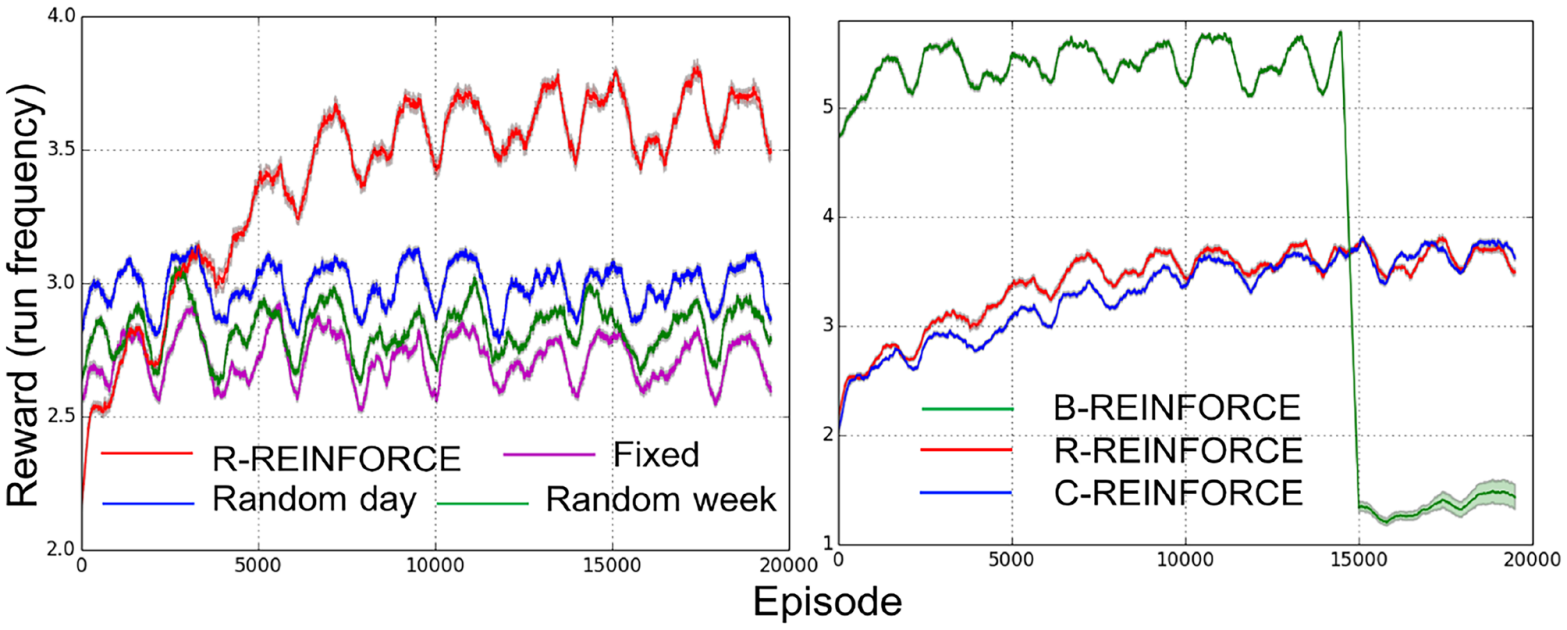
The simulation results shown the average reward of agents in the sliding windows of 500 episodes

The first experiment aims to examine whether the policy learned by our data-driven approach outperforms general rule-based policies (not considering the contextual information of users). We compared our RL-based agent (***R*** agent) with three baseline agents. All four agents send the same number of notifications per episode, but use different strategies. Three strategies of the baseline agents are (1) ‘random week agent’ sends 14 notifications randomly in each week; (2) ‘random day agent’ sends 2 notifications randomly in each day; (3) ‘fixed agent’ sends 2 notifications per day and they were evenly distributed (at 12:00 and 16:00). The performance of agents is shown in Fig. 3-left. We observed an obvious increase in the reward of ***R*** agent, while three others hold a relatively stable performance. It indicates our approach adaptively optimizes the policy to send a restricted amount of notifications with respect to user’s momentary context, and afterward outperforms all context-blind agents.

#### Evaluation of restricted policy

In the second experiment, we evaluated the efficiency of our restricted notification setting and how well the RL agents perform when incorporating this constraint during the learning in two different ways. One is applied and described in our RL algorithm of Reinforcement Learning Algorithm (***R*** agent). Second is to integrate it into the simulation environment: after the maximum number of notifications is reached in an episode, a notification will not be sent even if the algorithm decides to send one (***C*** agent). In Fig. 3-right, we found that although the ***R*** agent learns faster than the ***C*** agent (consistent with results shown in [32]), two agents show a similar performance after learning. In addition, we set up the ***B*** agent, which had no restriction on the number of notifications sent in each episode before 15,000 episodes. Afterwards, we integrated the restriction in its environment, leading to a dramatic performance drop in Fig. 3-right. This phenomenon demonstrates the different performances from an agent without restriction during learning (agent ***B***) and agents with restriction during learning (both the agent ***R*** and agent ***C***). It indicates that the policy learned without considering the restriction hardly performs well in a restricted mHealth setting, suggesting the importance of modeling this practical restriction in training RL algorithms.

#### Interpretation of learned policy

**Fig. 4:**
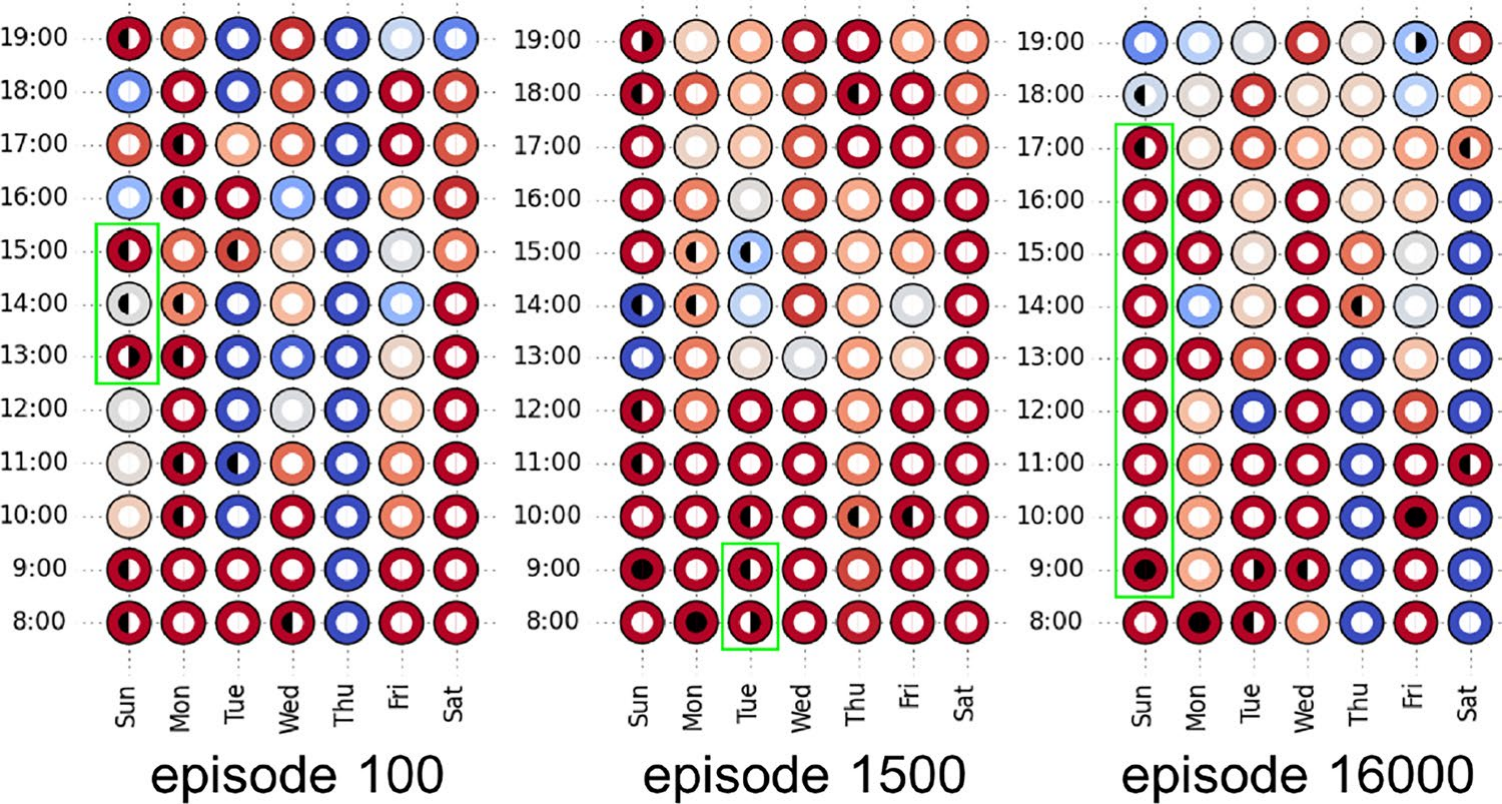
Information of three episodes in the ***R*** (R-REINFORCE) agent. Each circle represents one decision point, marked by hour and weekday. Black on the left side means ‘a notification’, and black on the right side means ‘a run’. The color of a circle represents the context desirability for running. While red and blue color correspond to the high and low desirability respectively, darker is more extreme

We further evaluated our approach by visualizing the detailed information of episodes in the learning process. Results of episode No. 100, 1500 and 16000 in a run of the ***R*** agent are presented in Fig. 4, which correspond to a policy before learning, a policy at the end of the first rapid learning process and a policy at the stable stage of learning in Fig. 3-left. We observed that at the beginning stage (episode 100), the ***R*** agent sends all notifications early in the episode. Afterwards, the agent learns to spread the restricted number of notifications over the entire episode (see episode 1500). This is the first strategy our agent learns, which leads to the first increase of the reward in Fig. 3. Moreover, the ***R*** agent learns to send notifications based on contextual situations. Notifications are sent in the decision points with very bad situations (dark blue ones) in the first two episodes, but almost all of them are sent under very good situations (dark red ones) in episode 16000. Finally, as indicated in green color in Fig. 4, the ***R*** agent realized that the simulated users are unlikely to run again in the hours following a recent run. Hence, the strategy of ‘not sending notification after a run’ seems to be learned.

## Conclusion and future work

In this paper, we explored the practical usage of adaptive and intelligent agents in personal mobile health intervention and developed an RL-based agent to optimize the strategy of adaptively delivering context-aware notifications. The simulation results showed that the policy learned by our RL agent is more efficient than manually defined strategies without context awareness. In particular, our work made two contributions to perform this practical learning task without bothering users too much. First, when incorporating prior knowledge from historical data and psychological theories for optimizing the policy, our proposed dynamic Bayes network can handle empirical data with various context space and flexible target activity. Second, we constrained notification frequency in a period and adapted an RL algorithm for this constraint. As far as we know, such constraint was never structurally studied and evaluated in a mHealth setting, our results provide evidence that it is essential to take the frequency restriction of certain actions into account in the learning process of RL. For future work, it would be interesting to examine the efficiency of various state-of-art RL algorithms considering this constraint. Also, the practical usage of our approach should be further evaluated in trials with real users. We have conducted a small-scale feasibility study [34]. Based on the initial results and learned lessons, we plan a longer study to evaluate the effectiveness of our pre-learned delivery strategy for comparable user groups.

## Supplementary information

Below is the link to the electronic supplementary material.

Supplementary file1 (PDF 316 KB)

## Footnotes

1 https://knmi.nl/nederland-nu/klimatologie/uurgegevens (last access on Oct 15th 2021)

2 https://github.com/sw1989/RLforPAUL

3 https://pytorch.org/

4 https://github.com/openai/gym

5 For details about the verification, see the supplementary information.

## Declarations

### Research involving human and animal participants

This article does not contain any studies with human participants or animals performed by any of the authors.
