# Supplementary material for: Optimizing Adaptive Notifications in Mobile Health Interventions Systems: Reinforcement Learning from a Data-driven Behavioral Simulator
Source: J Med Syst. 2021 Oct 18;45(12):102. doi: 10.1007/s10916-021-01773-0 (PMC8523513; doi:10.1007/s10916-021-01773-0)
Supplement: Supplementary file 1 — Supplementary file1 (PDF 316 KB) [file 10916_2021_1773_MOESM1_ESM.pdf]

Supplementary Information

for the paper 'Optimizing Adaptive Notifications in Mobile Health Interventions Systems: Reinforcement Learning from a Data-driven Behavioral Simulator'

A. User simulator verification

The model verification step served two purposes: (1) to verify whether the three cognitive parameters in the Dynamic Bayesian Network (DBN) would influence simulated running behavior as we intended in the design; and (2) to test whether a combination of theory-based plausible parameter values would produce realistic and sensible behavioral patterns.

In three simulations, we evaluated the influences of the three parameters in the DBN, namely memory retention rate σ, urge recovery rate μ and context desirability $\frac{P(C_t|A_t)}{P(C_t)}$. When one parameter was the target of evaluation in each round, te other two parameters were fixed their theory-based plausible values. We set σ at 0.8 based on [1] and μ at 0.05, which implies that 20 hours are needed for a person's running urge to fully recover. Context desirability was set at 1, representing neutral context that did not facilitate nor inhibit running behavior. The effects of these parameters were also examined with three different strategies of sending notifications. In the *fixed strategy*, two notifications were sent per day and they were evenly distributed across the day. In the *random-day strategy*, two notifications were sent per day but the exact hours were random. In the *random-week strategy*, 14 notifications were randomly sent over a week. In each simulation scenario, 100 users were simulated to make running decisions 12 times a day for 7 days. The simulated users were homogeneous, except that their initial memory accessibility of running were randomly drawn from a distribution based on its theoretical dynamics. We measured two outcome variables in Figure 1: the *average running frequency per person per day* and the *percentage of notification-contingent running relative to all running*.

Simulation results showed that, as expected, simulated users ran more frequently with higher σ, higher μ, and in more desirable context. It was also evident that sending two daily notifications at fixed or random moments motivated running better than send 14 notifications randomly over a week. Figure 1 (bottom) shows the same results for the outcome *percentage of notification-contingent running relative to all running.* Since notifications were supposed to only influence memory accessibility, it was not surprising that only memory retention rate had a negative effect on this outcome. This can be explained by the fact that if a user's memory decays slower, it is more likely that the user remembers and decides to run with some latency to the notifications.

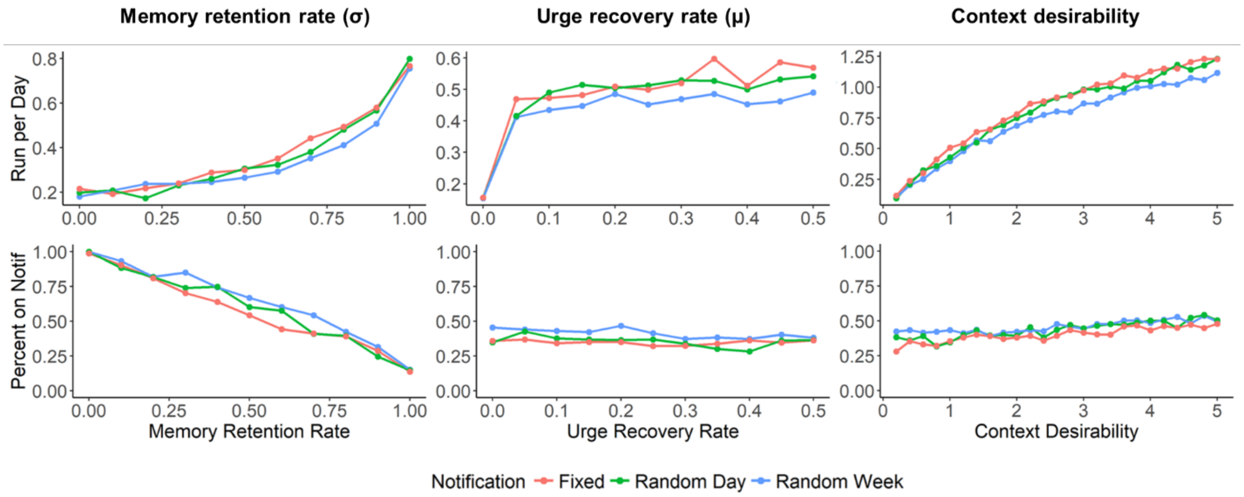

Fig. 1. Average running frequency per day (top) and percentage of running upon receiving notifications (bottom) as a function of notification strategy and varying parameters.

Overall, with memory retention rate σ at 0.8, urge recovery rate μ at 0.05, and a neutral context, simulated users would run approximately 0.5 times a day and about 30% of their decisions to run were made upon receiving notifications. Based on common sense, these outputs were realistic and reasonable. We thus fixed the memory and urge parameters in our user simulator and used them for the subsequent experiments.

References

[1] Robert Tobias. 2009. Changing behavior by memory aids: A social psychological model of prospective memory and habit development tested with dynamic field data. Psychological Review, 116, 2(2009), 408–438
